# Immunogenicity and safety assessment of a trivalent, inactivated split influenza vaccine in Korean children: Double-blind, randomized, active-controlled multicenter phase III clinical trial

**DOI:** 10.1080/21645515.2015.1017693

**Published:** 2015-04-15

**Authors:** Seung Beom Han, Jung-Woo Rhim, Hye Jo Shin, Soo Young Lee, Hyun-Hee Kim, Jong-Hyun Kim, Kyung-Yil Lee, Sang Hyuk Ma, Joon Soo Park, Hwang Min Kim, Chun Soo Kim, Dong Ho Kim, Young Youn Choi, Sung-Ho Cha, Young Jin Hong, Jin Han Kang

**Affiliations:** 1 Department of Pediatrics; The Catholic University of Korea College of Medicine; Seoul, Republic of Korea; 2 Department of Pediatrics; Changwon Fatima Hospital; Changwon, Republic of Korea; 3 Department of Pediatrics; Soonchunhyang University College of Medicine; Cheonan, Republic of Korea; 4 Department of Pediatrics; Yonsei University Wonju College of Medicine; Wonju, Republic of Korea; 5 Department of Pediatrics; Keimyung University College of Medicine; Daegu, Republic of Korea; 6 Department of Pediatrics; Korean Cancer Center Hospital; Seoul, Republic of Korea; 7 Department of Pediatrics; Chonnam National University Medical School; Gwangju, Republic of Korea; 8 Department of Pediatrics; Kyung Hee University School of Medicine; Seoul, Republic of Korea; 9 Department of Pediatrics; Inha University College of Medicine; Incheon, Republic of Korea

**Keywords:** child, clinical trial, influenza, Republic of Korea, vaccine

## Abstract

A multicenter, double-blind, randomized, active-control phase III clinical trial was performed to assess the immunogenicity and safety of a trivalent, inactivated split influenza vaccine. Korean children between the ages of 6 months and 18 y were enrolled and randomized into a study (study vaccine) or a control vaccine group (commercially available trivalent, inactivated split influenza vaccine) in a 5:1 ratio. Antibody responses were determined using hemagglutination inhibition assay, and post-vaccination immunogenicity was assessed based on seroconversion and seroprotection rates. For safety assessment, solicited local and systemic adverse events up to 28 d after vaccination and unsolicited adverse events up to 6 months after vaccination were evaluated. Immunogenicity was assessed in 337 and 68 children of the study and control groups. In the study vaccine group, seroconversion rates against influenza A/H1N1, A/H3N2, and B strains were 62.0% (95% CI: 56.8–67.2), 53.4% (95% CI: 48.1–58.7), and 54.9% (95% CI: 48.1–60.2), respectively. The corresponding seroprotection rates were 95.0% (95% CI: 92.6–97.3), 93.8% (95% CI: 91.2–96.4), and 95.3% (95% CI: 93.0–97.5). The lower 95% CI limits of the seroconversion and seroprotection rates were over 40% and 70%, respectively, against all strains. Seroconversion and seroprotection rates were not significantly different between the study and control vaccine groups. Furthermore, the frequencies of adverse events were not significantly different between the 2 vaccine groups, and no serious vaccination-related adverse events were noted. In conclusion, the study vaccine exhibited substantial immunogenicity and safety in Korean children and is expected to be clinically effective.

## Abbreviations

AE: adverse event
CI: confidence interval
FDA: Food and Drug Administration
GMR: geometric mean titer ratio
GMT: geometric mean titer
HI: hemagglutination inhibition
USA: United States of America
WHO: World Health Organization

## Introduction

The Advisory Committee on Immunization Practices of the United States of America (USA) recommends universal influenza vaccination for all children aged 6 months or more who do not have contraindications.^1^ The Korean Society of Pediatrics also expanded the influenza vaccine coverage from children with underlying diseases to all children aged between the ages of 6 and 59 months.^2^ This expansion of influenza vaccine coverage was based on the findings that the incidence, hospitalization rate, and mortality due to influenza were higher in young children than in adolescents and adults;^3-8^ young children contributed to the transmission of influenza in the family;^9^ and influenza vaccination in children decreased the incidences of influenza across the community including adults.^1,10,11^ Such an expansion of influenza vaccine coverage and the increased concern about the dangers of influenza after the pandemic in 2009, is expected to increase the demand for influenza vaccines. Considering that an influenza pandemic may recur, a stable supply of influenza vaccines at the national and regional levels should be ensured. Therefore, the World Health Organization (WHO) recommended increasing the production capacity for influenza vaccines, and in accordance with those recommendations, ILYANG Pharmaceutical Co., Ltd., one of Korean domestic pharmaceutical companies, developed a trivalent, inactivated split influenza vaccine. This egg-based vaccine was manufactured using chicken eggs, which were collected and transported to the manufacturing facility on the day they were laid. The eggs were fumigated using a vaporized hydrogen peroxide system and incubated for 10 d in a separated and well-controlled incubation area located in the manufacturing facility. This process decreased contamination of the eggs and increased vaccine-producing efficiency. In addition, a sucrose gradient purification system using a zonal centrifuge resulted in an increased influenza antigen purification rate. This study was conducted to evaluate the immunogenicity and safety of this egg-based, trivalent, inactivated split influenza vaccine in Korean children.

## Results

During the study period, 418 children were enrolled, and 416 of them were immunized with influenza vaccines. The study and control vaccine groups included 347 and 69 children, respectively. The distribution of gender and age was not significantly different between the 2 vaccine groups (**Table 1**). Among the vaccinated children, 405 (337 in the study vaccine group, 68 in the control vaccine group) completed the study according to the scheduled protocol. Immunogenicity assessment was performed for all of these 405 children. Safety assessment was performed for 416 vaccinated children.

**Table 1.** Gender and age distribution of enrolled children

	Study group	Control group	
Factor	(n = 347)	(n = 69)	*P* value
Gender, n (%)			0.3799^a^
Male	156 (45.0)	35 (50.7)	
Female	191 (55.0)	34 (49.3)	
Age, years, mean ± SD	7.8 ± 4.9	7.5 ± 5.0	0.5523^b^
Age group, n (%)			0.9363^a^
6 months - 3 y	68 (19.6)	14 (20.3)	
3 y – 9 y	130 (37.5	27 (39.1)	
9 y – 18 y	149 (42.9)	28 (40.6)	
Dose of vaccination, n (%)			0.9494^a^
One dose	316 (91.1)	63 (91.3)	
Two doses	31 (8.9)	6 (8.7)	

SD, standard deviation.

a calculated using a chi-square test.

b calculated using a Student's *t*-test.

### Immunogenicity in the study vaccine group

Among the 405 children assessed for immunogenicity, 370 (91.4%, 308 in the study vaccine group, 62 in the control vaccine group) with a history of influenza vaccination received a single dose of influenza vaccine, whereas the other 35 children (8.6%, 29 in the study vaccine group, 6 in the control vaccine group) with no history of influenza vaccination received 2 doses of influenza vaccine. The proportion of children who received 2 doses of vaccine was not significantly different between the 2 vaccine groups tested (**Table 1**).

In the study vaccine group, the pre-vaccination geometric mean titers (GMTs) of hemagglutination inhibition (HI) antibody against influenza A/H1N1, A/H3N2, and B strains were 24.4 (95% CI: 22.0–27.1), 31.9 (95% CI: 28.5–35.6), and 36.2 (95% CI: 32.6–40.2), respectively. The post-vaccination GMTs against influenza A/H1N1, A/H3N2, and B strains increased to 111.4 (95% CI: 101.5–122.3), 110.6 (95% CI: 100.9–121.2), and 130.5 (95% CI: 118.0–144.4), respectively (**Table 2**). As a result, geometric mean titer ratios (GMRs) for influenza A/H1N1, A/H3N2, and B strains were 4.6 (95% CI: 4.1–5.1), 3.5 (95% CI: 3.2–3.8), and 3.6 (3.3–4.0), respectively (**Table 2**). The corresponding seroconversion rates were 62.0% (209/337), 53.4% (180/337), 54.9% (185/337), respectively. The lower 95% CI limits of seroconversion rates against each influenza strain were all higher than 40% (**Table 2**). The seroprotection rates against influenza A/H1N1, A/H3N2, and B strains were 95.0% (320/337), 93.8% (316/337), and 95.3% (321/337), respectively (**Table 2**). The lower 95% CI limits of seroprotection rates against each influenza strain were all higher than 70% (**Table 2**). In addition, all age groups of the study vaccine group achieved the immunogenicity endpoints (**Table 2**). Children receiving either a single dose or 2 doses of influenza vaccine achieved the immunogenicity endpoints for seroconversion rates against all 3 influenza strains, but, not for seroprotection rates (**Table 3**).

**Table 2.** Immunogenicity of an influenza inactivated vaccine in children

	6 months – 3 years			3 years – 9 years		
	Study group	Control group		Study group	Control group	
Factor	(n = 65)	(n = 14)	*P* value	(n = 127)	(n = 27)	*P* value
A/H1N1						
Seroconversion rate, % (95% CI)	69.2 (58.0–80.5)	57.1 (31.2–83.1)	0.5314^a^	64.6 (56.3–72.9)	55.6 (36.8–74.3)	0.3785^b^
Seroprotection rate, % (95% CI)	81.5 (72.1–91.0)	64.3 (39.2–89.4)	0.1669^a^	97.6 (95.0–100.0)	96.3 (89.2–100.0)	0.5414^a^
GMT						
Pre-vaccination	14.3 (11.8–17.3)	13.7 (11.1–16.9)	0.3154^c^	26.4 (22.0–31.5)	30.3 (20.5–44.7)	<0.0001^c^
Post-vaccination	76.1 (59.4–97.4)	45.3 (28.3–72.5)	<0.0001^c^	125.0 (107.5–145.4)	92.7 (66.6–129.1)	<0.0001^c^
GMR	5.3 (4.2–6.7)	3.3 (2.0–5.5)	0.0078^c^	4.7 (4.0–5.7)	3.1 (2.1–4.4)	0.0035^c^
A/H3N2						
Seroconversion rate, % (95% CI)	69.2 (58.0–80.5)	50.0 (23.8–76.2)	0.2171^a^	48.8 (40.1–57.5)	63.0 (44.8–81.2)	0.1818^b^
Seroprotection rate, % (95% CI)	81.5 (72.1–91.0)	64.3 (39.2–89.4)	0.1669^a^	96.1 (92.7–99.5)	96.3 (89.2–100.0)	1.0000^a^
GMT						
Pre-vaccination	16.5 (12.3–22.3)	18.7 (8.7–39.9)	0.0373^c^	41.2 (35.1–48.3)	32.8 (20.5–52.4)	<0.0001^c^
Post-vaccination	94.2 (71.0–125.1)	69.5 (28.5–169.5)	<0.0001^c^	122.4 (106.5–140.7)	115.6 (81.4–164.2)	<0.0001^c^
GMR	5.7 (4.6–7.1)	3.7 (2.6–5.4)	0.0045^c^	3.0 (2.6–3.4)	3.5 (2.4–5.2)	0.2371^c^
B						
Seroconversion rate, % (95% CI)	58.5 (46.5–70.4)	64.3 (39.2–89.4)	0.6872^b^	59.1 (50.5–67.6)	74.1 (57.5–90.6)	0.1449^b^
Seroprotection rate, % (95% CI)	81.5 (72.1–91.0)	85.7 (67.4–100.0)	1.0000^a^	96.9 (93.8–99.9)	96.3 (89.2–100.0)	1.0000^a^
GMT						
Pre-vaccination	15.3 (12.9–18.1)	20.8 (14.1–30.5)	<0.0001^c^	30.7 (26.3–35.8)	33.0 (23.6–46.2)	<0.0001^c^
Post-vaccination	71.3 (54.8–92.8)	87.3 (41.2–184.7)	<0.0001^c^	126.4 (107.3–148.8)	141.1 (98.8–201.4)	<0.0001^c^
GMR	4.7 (3.6–6.0)	4.2 (2.2–7.9)	0.5819^c^	4.1 (3.5–4.9)	4.3 (2.9–6.3)	0.7819^c^
	**9 years – 18 years**			**Whole population**		
**Factor**	**Study group(n = 145)**	**Control group(n = 27)**	***P*** **value**	**Study group(n = 337)**	**Control group(n = 68)**	***P*** **value**
A/H1N1						
Seroconversion rate, % (95% CI)	56.6 (48.5–64.6)	55.6 (36.8–74.3)	0.9236^b^	62.0 (56.8–67.2)	55.9 (44.1–67.7)	0.3441^b^
Seroprotection rate, % (95% CI)	98.6 (96.7–100.0)	96.3 (89.2–100.0)	0.4028^a^	95.0 (92.6–97.3)	89.7 (82.5–96.9)	0.1528^a^
GMT						
Pre-vaccination	29.0 (24.9–33.7)	27.6 (18.6–41.0)	0.0122^c^	24.4 (22.0–27.1)	24.8 (19.8–31.1)	0.2527^c^
Post-vaccination	119.5 (105.4–135.5)	119.4 (81.2–175.4)	0.7651^c^	111.4 (101.5–122.3)	88.5 (70.3–111.2)	<0.0001^c^
GMR	4.1 (3.5–4.9)	4.3 (2.7–6.9)	0.7372^c^	4.6 (4.1–5.1)	3.6 (2.8–4.6)	0.0058^c^
A/H3N2						
Seroconversion rate, % (95% CI)	50.3 (42.2–58.1)	55.6 (36.8–74.3)	0.6190^b^	53.4 (48.1–58.7)	55.6 (45.6–69.1)	0.5520^b^
Seroprotection rate, % (95% CI)	97.2 (94.6–99.9)	100.0 (100.0–100.0)	1.0000^a^	93.8 (91.2–96.4)	91.2 (84.4–97.9)	0.4265^a^
GMT						
Pre-vaccination	34.1 (29.1–40.0)	35.1 (23.2–53.3)	0.0806^c^	31.9 (28.5–35.6)	30.0 (22.6–39.8)	<0.0001^c^
Post-vaccination	108.7 (96.3–122.8)	117.9 (88.8–156.5)	<0.0001^c^	110.6 (100.9–121.2)	104.9 (82.3–133.8)	<0.0001^c^
GMR	3.2 (2.7–3.7)	3.4 (2.2–5.2)	0.7628^c^	3.5 (3.2–3.8)	3.5 (2.8–4.4)	0.9273^c^
B						
Seroconversion rate, % (95% CI)	46.7 (41.5–57.8)	59.3 (40.7–77.8)	0.3593^b^	54.9 (48.1–60.2)	66.2 (54.9–77.4)	0.0867^b^
Seroprotection rate, % (95% CI)	100.0 (100.0–100.0)	100.0 (100.0–100.0)	NA	95.3 (93.0–97.5)	95.6 (90.7–100.0)	1.0000^a^
GMT						
Pre-vaccination	61.6 (53.8–70.6)	65.7 (50.0–86.3)	<0.0001^c^	36.2 (32.6–40.2)	39.4 (32.0–48.7)	<0.0001^c^
Post-vaccination	176.1 (155.8–199.0)	217.3 (160.7–293.8)	<0.0001^c^	130.5 (118.0–144.4)	151.7 (119.3–192.9)	<0.0001^c^
GMR	2.9 (2.5–3.3)	3.3 (2.2–5.0)	0.3709^c^	3.6 (3.3–4.0)	3.9 (3.0–4.9)	0.4633^c^

CI, confidence interval; GMT, geometric mean titer; GMR, geometric mean titer ratio; NA, not available.

a calculated using a Fisher's exact test.

b calculated using a chi-square test.

c calculated using a Student's *t*-test.

**Table 3.** Immunogenicity according to the dose of influenza vaccination

	One dose			Two doses		
	Study group	Control group		Study group	Control group	
Factor	(n = 308)	(n = 62)	*P* value	(n = 29)	(n = 6)	*P* value
A/H1N1						
Seroconversion rate, % (95% CI)	60.1 (54.6–65.5)	54.8 (42.5–67.2)	0.4449^a^	82.8 (69.0–96.5)	66.7 (29.0–100.0)	0.5762^b^
Seroprotection rate, % (95% CI)	96.1 (93.9–98.3)	91.9 (85.2–98.7)	0.1781^b^	82.8 (69.0–96.5)	66.7 (29.0–100.0)	0.5762^b^
GMT						
Pre-vaccination	26.7 (24.0–29.7)	26.7 (21.0–34.0)	0.9788^c^	9.4 (7.5–11.7)	11.5 (9.2–14.3)	0.0109^c^
Post-vaccination	115.1 (104.6–126.6)	94.1 (74.1–119.6)	<0.0001^c^	78.8 (54.2–114.4)	46.7 (20.3–107.4)	<0.0001^c^
GMR	4.3 (3.9–4.8)	3.5 (2.7–4.6)	0.0349^c^	8.4 (5.8–12.3)	4.1 (1.7–9.9)	0.0009^c^
A/H3N2						
Seroconversion rate, % (95% CI)	51.3 (45.7–56.9)	56.5 (44.1–68.8)	0.4587^a^	75.9 (60.3–91.4)	66.7 (29.0–100.0)	0.6353^b^
Seroprotection rate, % (95% CI)	95.5 (93.1–97.8)	93.6 (87.4–99.7)	0.5185^b^	75.9 (60.3–91.4)	66.7 (29.0–100.0)	0.6353^b^
GMT						
Pre-vaccination	33.9 (30.3–37.9)	31.2 (23.2–42.1)	<0.0001^c^	16.4 (10.1–26.6)	20.0 (5.9–67.6)	0.0298^c^
Post-vaccination	111.2 (101.8–121.4)	104.6 (82.4–132.9)	<0.0001^c^	104.7 (61.4–178.5)	107.8 (18.9–616.8)	0.1124^c^
GMR	3.3 (3.0–3.6)	3.4 (2.6–4.3)	0.8358^c^	6.4 (4.5–9.0)	5.4 (2.7–10.7)	0.3668^c^
B						
Seroconversion rate, % (95% CI)	54.2 (48.7–59.8)	66.1 (54.4–77.9)	0.0846^a^	62.1 (44.4–79.7)	66.7 (29.0–100.0)	1.0000^b^
Seroprotection rate, % (95% CI)	96.8 (94.8–98.7)	96.8 (92.4–100.0)	1.0000^b^	79.3 (64.6–94.1)	96.8 (92.5–100.0)	1.0000^b^
GMT						
Pre-vaccination	40.1 (36.1–44.5)	43.0 (34.6–53.4)	<0.0001^c^	12.4 (9.7–15.7)	16.2 (10.3–25.5)	<0.0001^c^
Post-vaccination	137.2 (124.0–151.6)	166.6 (131.1–211.6)	<0.0001^c^	77.2 (48.0–124.2)	57.7 (18.8–176.7)	<0.0001^c^
GMR	3.4 (3.1–3.8)	3.9 (3.0–5.0)	0.1847^c^	6.2 (4.0–9.8)	3.6 (1.4–9.4)	0.0665^c^

CI, confidence interval; GMT, geometric mean titer; GMR, geometric mean titer ratio.

a calculated using a chi-square test.

b calculated using a Fisher's exact test.

c calculated using a Student's *t*-test.

### Comparison of immunogenicity between the study and control groups

The seroconversion and seroprotection rates against all 3 influenza strains were not significantly different between the study and control vaccine groups (**Table 2**). Within each age group, the seroconversion and seroprotection rates were not significantly different between the 2 vaccine groups (**Table 2**). The upper 95% CI limits of HI antibody GMTs in the study vaccine group were less than 1.5-fold of those in the control vaccine group for all 3 influenza strains. However, the differences for the upper 95% CI limits of seroconversion rates between the study and control vaccine groups were more than 10% for influenza A/H3N2 and influenza B strains.

### Subgroup analysis of the immunogenicity in children without protective HI antibody titers prior to vaccination

For each influenza virus strain, the number of children without prior protective immunity against influenza (HI antibody titer <1:40) was between 16 (4.7%) and 21 (6.2%) in the study group and between 3 (4.4%) and 7 (10.3%) in the control group (**Table 4**). Of the 3 age groups, the proportion of children without prior protective immunity was highest in the children aged 6 months to 3 y accounting for 18.5% of the study group and 14.3 to 35.7% of the control group for each influenza virus strain (**Table 4**). Seroconversion rates for these children were between 0.0% and 61.9% without statistically significant difference between the study and control vaccine groups (**Table 4**).

**Table 4.** Seroconversion rates in children without protective immunity against influenza prior to vaccination

	6 months – 3 years			3 years – 9 years		
Factor	Study group(n = 65)	Control group(n = 14)	*P* value^a^	Study group(n = 127)	Control group(n = 27)	*P* value^a^
A/H1N1 Number of subjects Seroconversion rate, % (95% CI)	12 25.0 (0.5–49.5)	5 0.0 (0.0–0.0)	0.5147	3 66.7 (13.3–100.0)	1 100.0 (100.0–100.0)	1.0000
A/H3N2 Number of subjects Seroconversion rate, % (95% CI)	12 75.0 (50.5–99.5)	5 60.0 (17.1–100.0)	0.6	5 80.0 (44.9–100.0)	1 0.0 (0.0–0.0)	0.3333
B Number of subjects Seroconversion rate, % (95% CI)	12 16.7 (0.0–37.8)	2 0.0 (0.0–0.0)	1.0000	4 25.0 (0.0–67.4)	1 0.0 (0.0–0.0)	1.0000
**Factor**	**9 years – 18 years**			**Whole population**		
**Study group (n = 145)**	**Control group (n = 27)**	***P* value^a^**	**Study group (n = 337)**	**Control group (n = 68)**	***P* value^a^**
A/H1N1 Number of subjects Seroconversion rate, % (95% CI)	2 50.0 (0.0–100.0)	1 100.0 (100.0–100.0)	1.0000	17 35.3 (12.6–58.0)	7 28.6 (0.0–62.0)	1.0000
A/H3N2 Number of subjects Seroconversion rate, % (95% CI)	4 0.0 (0.0–0.0)	0 NA	NA	21 61.9 (41.1–82.7)	6 50.0 (10.0–90.0)	0.6618
B Number of subjects Seroconversion rate, % (95% CI)	0 NA	0 NA	NA	16 18.8 (0.0–37.9)	3 0.0 (0.0–0.0)	1.0000

CI, confidence interval; NA, not available.

a calculated using a Fisher's exact test.

### Safety

Eight hundred forty-five adverse events (AEs) in 292 (70.2%) children were reported within 28 d after vaccination. These consisted of 710 episodes in 239 (68.9%) children of the study vaccine group and 135 episodes in 53 (76.8%) children of the control vaccine group (*P* = 0.1881). Six months after vaccination, 851 AEs were reported in 294 (70.7%) children. These consisted of 716 episodes in 241 (69.5%) children of the study vaccine group and 135 episodes in 53 (76.8%) children of the control vaccine group (**Table 5**). The incidences of total, solicited local and systemic, and unsolicited AEs were not significantly different between the study and control vaccine groups (**Table 5**). Local tenderness was the most frequent form of solicited local AE while malaise was the most frequent form of solicited systemic AE in both vaccine groups (**Table 6**). One hundred fifty-six episodes of unsolicited AEs in 92 (26.5%) children of the study vaccine group, and 29 episodes of unsolicited AEs in 19 (27.5%) children of the control vaccine group were reported 6 months after vaccination. Upper respiratory infections (63.8%) were the most frequent among these reports. Three episodes of serious AEs in 3 (0.9%) children of the study vaccine group were reported within 28 d after vaccination: 2 cases of acute otitis media and one case of febrile seizures. Between 28 d and 6 months after vaccination, additional 6 episodes of serious AEs were reported in 5 (1.4%) children of the study vaccine group: 2 cases of bronchopneumonia and one case each of acute bronchiolitis, acute gastroenteritis, gross hematuria, and Kawasaki disease. There were no serious AEs in the control vaccine group. All of the reported serious AEs were considered unrelated to influenza vaccination.

**Table 5.** Frequencies of adverse events reported within 6 months after influenza vaccination

Factor	6 month – 3 years			3 years – 9 years		
Study group (n = 68)	Control group (n = 14)	*P* value	Study group (n = 130)	Control group (n = 27)	*P* value
Total adverse events	45 (66.2)	12 (85.7)	0.2080^a^	96 (73/8)	20 (74.1)	0.9804^b^
Solicited local adverse events	20 (29.4)	7 (50.0)	0.2100^a^	74 (56.9)	17 (63.0)	0.5629^b^
Solicited systemic adverse events	14 (20.6)^a^	7 (50.0)	0.0393^a^	38 (29.2)	9 (33.3)	0.6719^b^
Unsolicited adverse events	37 (54.4)	9 (64.3)	0.4978^b^	43 (33.1)	7 (25.9)	0.4680^b^
Serious adverse events	5 (7.4)	0 (0.0)	0.5821^a^	3 (2.3)	0 (0.0)	1.0000^a^
**Factor**	**9 years – 18 years**			**Whole population**		
**Study group (n = 149)**	**Control group (n = 28)**	***P*** **value**	**Study group (n = 347)**	**Control group (n = 69)**	***P*** **value**
Total adverse events	100 (67.1)	21 (75.0)	0.4104^b^	241 (69.5)	53 (76.8)	0.2201^b^
Solicited local adverse events	91 (61.1)	17 (60.7)	0.9714^b^	185 (53.3)	41 (59.4)	0.3524^b^
Solicited systemic adverse events	56 (37.6)	9 (32.1)	0.5837^b^	108 (31.1)	25 (36.2)	0.4060^b^
Unsolicited adverse events	12 (8.1)	3 (10.7)	0.7099^a^	92 (26.5)	19 (27.5)	0.8607^b^
Serious adverse events	0 (0.0)	0 (0.0)	NA	8 (2.3)	0 (0.0)	0.3626^a^

Data are numbers (%) of subjects who experienced adverse events.

NA, not available.

a calculated using a Fisher's exact test.

b calculated using a chi-square test.

**Table 6.** Episodes of solicited adverse events within 6 months after influenza vaccination

	Study group (n = 347)		Control group (n = 69)	
Factor	Total	≥grade 2	Total	≥grade 2
Solicited local adverse events				
Tenderness	169	44 (26.0)	36	7 (19.4)
Pain	120	4 (3.3)	26	0 (0.0)
Erythema	48	8 (16.7)	5	0 (0.0)
Swelling	19	2 (10.5)	2	0 (0.0)
Solicited systemic adverse events				
Myalgia	67	16 (23.9)	8	0 (0.0)
Malaise	57	13 (22.8)	12	7 (58.3)
Headache	34	9 (26.5)	7	1 (14.3)
Fever	25	10 (40.0)	6	5 (83.3)
Nausea/vomiting	13	2 (15.4)	2	0 (0.0)
Diarrhea	8	0 (0.0)	2	0 (0.0)

Data are numbers (%) of adverse events.

## Discussion

The present study was performed to evaluate the immunogenicity and safety of an influenza vaccine manufactured by a Korean pharmaceutical company. This vaccine was produced in a well-controlled incubation facility using fumigation system for the eggs and an antigen purification system, resulting in an increased purification rate. This vaccine achieved the immunogenicity endpoints recommended by the USA Food and Drug Administration (FDA),^12^ and showed similar immunogenicity to previously studied inactivated influenza vaccines in children.^13-21^

Studies on the clinical efficacy and effectiveness of inactivated influenza vaccines in children are scarce, and several previous studies reported relatively poorer immunogenicity of inactivated influenza vaccines in young children than in older children and adults.^20-23^ Therefore, some experts disagree with universal influenza vaccination for infants and young children.^24^ However, recent studies reported sufficient effectiveness of influenza vaccines in children younger than 2 or 3 y of age,^25,26^ and the present study showed sufficient seroprotection rates after vaccination even in children younger than 3 y. If we use sufficiently immunogenic influenza vaccines in young children such as this study vaccine and maintain appropriate methods for vaccine transport and storage, sufficient clinical efficacy of influenza vaccines can be achieved even in infants and young children.

Children without protective HI antibody titer prior to vaccination in the present study showed lower seroconversion rates than previously reported results.^19,20^ Their seroconversion rates in the control vaccine group were lower than those in the previous studies using the same influenza vaccines as in the control vaccine group of the present study.^15,19^ These results were assumed to be caused by the small number of enrolled children who did not have prior protective immunity in the present study. The proportion of children without prior protective immunity was only 4.7 to 6.2% in the study vaccine group and 4.4 to 10.3% in the control vaccine group for each influenza virus strain. For an accurate assessment of immunogenicity in children without prior protective immunity, further studies including more children should be performed. It may be useful to perform HI antibody tests before enrollment to screen for children without prior protective immunity. In addition, conducting vaccine studies before influenza season, i.e. summer or early fall, may prevent children from being exposed to influenza virus in the community during influenza season and naturally acquiring protective immunity. These strategies may help include more children without prior protective immunity against influenza.

The USA FDA proposed endpoints for determining the non-inferiority immunogenicity of an influenza vaccine in comparison with that of a licensed vaccine: the ratio of the upper limits of the 2-sided 95% CIs of GMTs of the 2 vaccines (GMT of a licensed vaccine/GMT of a new vaccine) should be <1.5 and the difference between the upper limits of the 2-sided 95% CIs of seroconversion rates of the 2 vaccines (seroconversion rate of a licensed vaccine – seroconversion rate of a new vaccine) should be <10%.^12^ Although the study vaccine in the present study achieved the immunogenicity endpoints, it did not achieve the non-inferiority endpoints. While the ratio of GMTs was less than 1.5, the difference for seroconversion rates was higher than 10% for influenza A/H3N2 and B strains. These results were assumed to be caused by the small number of enrolled subjects, especially in the control vaccine group. A further study including an appropriate number of subjects in both the study and control vaccine groups should be performed to assess the non-inferiority of the study vaccine in comparison with that of a licensed vaccine.

Immunogenicity against the influenza B strain was lower than that against the influenza A strains in the present study. This discrepancy was also reported in previous vaccine studies.^14,17,19,27,28^ Accordingly, efficacy of the vaccine against the influenza B strain was lower than that against influenza A strains.^27^ These results were thought to be caused by the relatively lower immunogenicity of influenza B antigens or the relatively lower sensitivity of the HI assay for influenza B as compared to influenza A.^28^ Efforts to improve vaccine immunogenicity against influenza B should be made in order to improve vaccine efficacy against this strain. If we consider that 2 lineages of the influenza B strain have been co-circulating in the community,^29,30^ vaccinations with quadrivalent influenza vaccines containing both the lineages may be necessary to improve vaccine efficacy against influenza B. Several studies have reported sufficient immunogenicity and safety of quadrivalent inactivated and quadrivalent live-attenuated influenza vaccines in children.^31-33^

The USA FDA recommends assessing the safety of influenza vaccines until 6 months after vaccination, although this practice is not obligatory.^12^ However, only a few studies on influenza vaccines in children evaluated their safety for this length of time.^14^ In the present study, we observed AEs until 6 months after influenza vaccination, and there were no vaccine-related serious AEs during the observation. In addition, there was no significant difference between the study vaccine and a commercially available influenza vaccine in terms of the incidence of AEs. One child in the study vaccine group experienced febrile seizures after vaccination. The fever developed 5 d after vaccination, and the seizures developed one day after the fever. This child had a previous history of febrile seizures, and recovered without any sequelae. More information on AEs will be collected through post marketing surveillance.

This study has several limitations. As mentioned above, the number of children enrolled in the control vaccine group in this study was too small to establish non-inferiority immunogenicity of the study vaccine compared to a commercially available vaccine. In addition, the number of children without prior protective immunity on enrollment was too small to determine their exact seroconversion and seroprotection rates. Future studies should include more children, especially children who are younger than 3 y and have no prior history of influenza vaccination. Clinical efficacy and effectiveness of vaccines may not be proportional to the immunogenicity identified through serologic tests. In particular, studies on the clinical efficacy and effectiveness of influenza vaccines should not be limited to one year but should be performed over several years because circulating influenza viral strains can be different every year. A long-term study on the clinical efficacy and AEs of the study vaccine should be performed to support its clinical usefulness.

In conclusion, the study vaccine exhibited sufficient immunogenicity and was deemed safe to use in children. Future studies will be performed to assess its clinical efficacy and effectiveness against influenza and to survey the development of AEs.

## Materials and Methods

### Study design and subjects

A double-blind randomized active-control multicenter phase III clinical trial was performed to evaluate the immunogenicity and safety of a trivalent, inactivated split influenza vaccine. Healthy children between 6 months and 18 y of age were enrolled between October 2013 and January 2014. They were divided into three groups according to their age: 6 months to <3 y, 3 y to <9 y, and 9 y to <18 y. In each age group, the enrolled children were randomized in a 5:1 ratio to receive either the study influenza vaccine or the control influenza vaccine. The following children were excluded from this study: children who were hypersensitive to any vaccine components including egg proteins, had Down syndrome, a cytogenetic disorder, a bleeding tendency, or any other chronic underlying disorders, or had a previous history of an immunodeficiency or Guillain-Barré syndrome. Children who had received immune suppressants, blood products or intravenous immunoglobulins within the past 3 months, received glucocorticoids (≥2 mg/kg/day as prednisolone) for more than 2 weeks, had a febrile illness within the past 3 days, received an influenza vaccine within the past 6 months, or received any other vaccines within the past one month were also excluded. This study was approved by the Institutional Review Board of each participating hospital. Informed consent was obtained from enrolled children (older than 7 or 10 y of age depending on the institution) and their parents.

### Vaccines and vaccination

The study vaccine was an egg-based, trivalent, inactivated split influenza vaccine, IL-YANG FLU Vaccine Prefilled Syringe INJ (ILYANG Pharmaceutical Co., Ltd., Seoul, Korea). The control vaccine was a commercially available trivalent inactivated split influenza vaccine, VAXIGRIP® (Sanofi Pasteur SA, Lyon, France). Each 0.5 mL of the study and control vaccines contained 15 μg of hemagglutinin antigen of each of the 3 influenza strains recommended for the 2013–2014 northern hemisphere influenza season by the WHO. Accordingly, the study vaccine contained influenza A/California/7/2009 NYMC X-181 (H1N1), A/Texas/50/2012 NYMC X-223 (H3N2), and B/Massachusetts/2/2012 NYMC BX-51B, and the control vaccine contained influenza A/California/7/2009 NYMC X-179A (H1N1), A/Texas/50/2012 NYMC X-223A (H3N2), and B/Massachusetts/2/2012 NYMC BX-51B. Children younger than 3 y, and those aged 3 y or more received 0.25 mL and 0.5 mL of influenza vaccine, respectively. Children aged younger than 9 y of age with no history of influenza vaccination received 2 doses at a 4-week interval.

### Assessment of immunogenicity

For assessing immunogenicity, 3–5 mL of blood was collected from each child immediately before and 28 d after each vaccination. Antibody titers against hemagglutinins of each vaccine strain were determined using a HI assay. HI assays were performed according to the previously reported methods at the Vaccine Bio Research Institute, College of Medicine, the Catholic University of Korea, Seoul, Republic of Korea.^34^ Seroconversion was defined as follows: when the HI antibody titer before vaccination was <1:10 and the titer after vaccination was ≥1:40, or when the HI antibody titer before vaccination was ≥1:10 and the titer increased 4-fold or more after vaccination.^12^ Seroprotection was defined as when the post-vaccination HI antibody titer was ≥1:40.^12^ HI antibody titers lower than the threshold level (1:10) were assigned to be 1:5, and GMTs of HI antibody titers before and after vaccination in each vaccine group were calculated. The GMR was defined as the ratio of the post-vaccination GMT of HI antibody titers to the pre-vaccination GMT of HI antibody titers. Immunogenicity was evaluated in accordance with the USA FDA guidance for clinical data needed to support the licensure of seasonal inactivated influenza vaccines using seroconversion and seroprotection rates.^12^ The FDA guidance defines the immunogenicity endpoints as the follows: the lower limit of the 2-sided 95% CI for the seroconversion rates should be ≥40 % and the lower limit of the 2-sided 95% CI for the seroprotection rate should be ≥70 %.^12^

### Assessment of safety

To assess the safety of the study vaccine, participants were contacted by telephone within a week after vaccination, and visited the participated hospitals 28 d and 6 months after vaccination. Solicited local AEs included pain, tenderness, erythema, and swelling. Solicited systemic AEs included fever, nausea/vomiting, diarrhea, headache, malaise, and myalgia. Serious AEs were defined as any life-threatening or fatal events, and any events causing hospitalization or permanent functional disabilities. Any solicited and unsolicited AEs occurring up to 28 d after vaccination were to be recorded in a provided diary. Any unsolicited AEs occurring up to 6 months after vaccination were to be recorded in the same diary. Subjects were directed to report any serious AEs occurring up to 6 months after vaccination immediately to the investigators. AEs were graded as follows: grade 0, no symptoms, erythema and swelling <2.5 cm, or elevated body temperature <37.5°C; grade 1, symptoms not-restricting activities, erythema and swelling of 2.5–5.0 cm, or elevated body temperature of 37.5–37.9°C; grade 2, symptoms restricting activities, erythema and swelling of 5.0–10.0 cm, or fever of 38.0–38.4°C; grade 3, symptoms restricting routine activities, erythema and swelling ≥10.0 cm, or fever of 38.5–39.0°C; grade 4, symptoms causing medical examinations, or fever >39.0°C.

### Statistical analysis

The sample size was calculated based on the statistical power required to meet the immunogenicity endpoints. Two hundred ninety-six subjects of the study vaccine group were required to achieve a seroconversion rate over 40% with an assumption of the seroconversion rate of the study vaccine to be 50% and an overall power of 80%. With an attrition rate of 10%, 329 subjects were required in the study vaccine group. The number of subjects in the control vaccine group was decided to be 66 according to the 5:1 ratio. Safety was assessed in all children who received at least one vaccination, and immunogenicity was assessed in children who completed this study according to the scheduled study protocol. Numerical factors such as GMTs and GMRs of the study and control vaccine groups were compared using a Student's *t*-test. Categorical factors such as seroconversion rate, seroprotection rate, and incidences of AEs were compared using a chi-square test or Fisher's exact test. Statistical analyses were performed with SPSS Statistics 17.0 (SPSS Inc., Chicago, IL, USA), and statistical significance was defined as a 2-tailed *P* value < 0.05.
